# EXAMINING THE PRELIMINARY EFFECTIVENESS OF THE SENIOR COMPANION PROGRAM PLUS FOR AFRICAN AMERICAN CAREGIVERS

**DOI:** 10.1093/geroni/igad104.1085

**Published:** 2023-12-21

**Authors:** Ling Xu, Noelle Fields, Ishan Williams, Joseph Gaugler, Alan Kunz Lomelin, Daisha Cipher, Gretchen Feinhals

**Affiliations:** The University of Texas at Arlington, Arlington, Texas, United States; The University of Texas at Arlington, Arlington, Texas, United States; University of Virginia, Charlottesville, Virginia, United States; University of Minnesota, Minneapolis, Minnesota, United States; University of Texas at Arlington, Arlington, Texas, United States; The University of Texas at Arlington, Arlington, Texas, United States; The Senior Source, Dallas, Texas, United States

## Abstract

**Objectives:**

A culturally informed, peer-led, lay provider model, the Senior Companion Program (SCP) Plus, was implemented to decrease caregiving burden/stress and improve coping skills, and social support for African American ADRD caregivers. This study reported the preliminary effectiveness of this intervention.

**Methods:**

An explanatory sequential mixed methods design was used in this study and a randomized control trial was conducted for the SCP Plus intervention among participants in three sites (N = 20). A sub-sample of participants (n = 7) consented to a qualitative interview about their experiences with the intervention. The Wilcoxon signed-rank tests, Friedman tests, and one-way repeated measures ANOVA were conducted for quantitative analyses. Thematic analysis was used for the qualitative interviews.

**Results:**

Results demonstrated that knowledge of AD/dementia (KAD) and preparedness for caregiving were significantly improved for all senior companions in the intervention group. Results also showed that caregivers in the intervention group reported significantly decreased caregiving burden, as well as increased KAD, satisfaction with social support, and positive aspects of caregiving. Themes from the qualitative interviews included: learning new skills about caregiving, gaining knowledge about ADRD, and benefits for the dyad. Discussions: Findings from this study implied that SCP Plus was a promising model for African American family caregivers as it benefits both the SC volunteers and the African American ADRD family caregivers.

